# Immune thrombocytopenia possibly triggered by multiple tick bites

**DOI:** 10.1590/S1678-9946202567001

**Published:** 2025-01-20

**Authors:** Carlos Ramiro Silva-Ramos, Andrés Eduardo Prieto-Torres, Abraham Katime Zuñiga, Jesús A. Cortés-Vecino, Bertha Lacouture Ortiz, Constanza Cuellar, Leidy J. Medina-Lozano, Álvaro A. Faccini-Martínez

**Affiliations:** 1 Pontificia Universidad Javeriana, Facultad de Ciencias, Grupo de Enfermedades Infecciosas, Bogotá, Colombia; 2 Hospital Militar Central, Servicio de Medicina Interna, Bogotá, Colombia; 3 Clínica El Prado, Servicio de Infectología, Santa Marta, Magdalena, Colombia; 4 Universidad del Magdalena, Facultad de Medicina, Santa Marta, Magdalena, Colombia; 5 Universidad Nacional de Colombia, Facultad de Medicina Veterinaria y de Zootecnia, Departamento de Salud Animal, Laboratorio de Parasitología Veterinaria, Bogotá, Colombia; 6 Vigilancia en Salud Pública del Departamento del Magdalena, Santa Marta, Magdalena, Colombia; 7 Hospital Militar Central, Servicio de Infectología, Bogotá, Colombia; 8 Universidad Militar Nueva Granada, Facultad de Medicina, Bogotá, Colombia

**Keywords:** Purpura, Thrombocytopenic, Idiopathic, Ticks, Colombia

## Abstract

Immune thrombocytopenia (ITP) is an autoimmune hematological condition characterized by a markedly isolated decrease in platelets without any apparent associated clinical conditions, resulting in bleeding and bruising of the skin, mucous membranes, and major organs. It is often triggered by preceding illness or several immune stimulants such as immunizations, infections, allergic reactions, among others. While uncommon, arthropod bites can trigger acute ITP. Four cases have been reported due to bee stings and insect bites, as well as a case of ITP following honeybee-venom therapy. Here, we report a case of acute ITP possibly triggered by multiple tick bites.

## INTRODUCTION

Immune thrombocytopenia (ITP) is an autoimmune hematological condition characterized by a markedly isolated decrease in platelets without any apparent associated clinical conditions, resulting in bleeding and bruising of skin, mucous membranes, and major organs^1^. Acute ITP is more common, typically lasts less than six months and usually affects children, whereas chronic ITP typically affects adults and is often triggered by preceding illness or several immune stimulants such as immunizations, infections, allergic reactions, among others^1^. Here, we report a case of acute ITP possibly triggered by multiple tick bites.

## CASE REPORT

**Figure 1:**
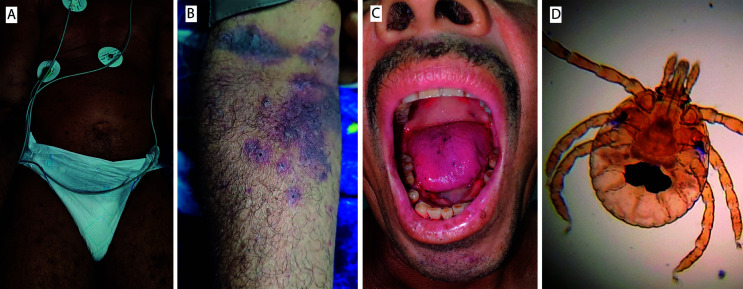
- Immune thrombocytopenia possibly triggered by multiple tick bites in a Colombian patient: (A) Multiple disseminated cutaneous crusted lesions by tick bites; (B) Hematoma on the right arm and crusted lesions by tick bites; (C) Oral purpura and gingival bleeding; (D) *Amblyomma* spp. larvae collected from the pastures where the patient worked.

On January 14, 2024, a previously healthy 61-year-old male from the rural region of Santa Rosa de Lima, Magdalena department, Colombia, was referred to our institution in Santa Marta city due to a two-day history of profound gingival bleeding associated with severe thrombocytopenia (16,000/µL) without fever. The patient reported multiple tick bites acquired during agricultural work in pastures in the three days before symptom onset. Physical examination was remarkable for mucous and cutaneous pallor, approximately 80 disseminated cutaneous crusted lesions, a hematoma on the right arm, oral purpura, and gingival bleeding (Figures 1A, 1B, and 1C). Laboratory results revealed severe thrombocytopenia, eosinophilia, and slightly elevated creatinine (Table 1). Consequently, he received two platelet transfusions, methylergotamine, tranexamic acid, and vitamin K, and was transferred to the intensive care unit (ICU). All other parameters were within normal ranges, and an abdominal ultrasound revealed no abnormalities. Additional investigations were conducted to identify an etiology. Serologic tests for dengue, *Leptospira*, HIV, HBV, and syphilis yielded negative results. His IgE value was slightly elevated, but other blood tests for autoimmune disease were normal (Table 1).

**Table 1:** - Laboratory parameters from the reported case, January 14 to January 19, 2024.

Parameter	January 14^th^	January 17^th^	January 19^th^	Reference
Leukocytes	8.48 10^9/L	20.66 10^9/L	23.94 10^9/L	4 – 10
Neutrophils	5.35 10^9/L	18.24 10^9/L	20.34 10^9/L	2 – 7
Lymphocytes	1.47 10^9/L	1.24 10^9/L	-	0.8 – 4
Eosinophils	0.96 10^9/L	0.0 10^9/L	-	0.02 – 0.5
Basophils	0.02 10^9/L	0.02 10^9/L	-	0 – 0.1
Monocytes	0.68 10^9/L	1.23 10^9/L	-	0.12 – 1.2
Hemoglobin	13.8 g/dL	8.5 g/dL	7.7 g/dL	12 – 16
Hematocrit	40 %	24.4%	21%	40 – 54
MCV	82.1 fL	82.8 fL	-	80 – 100
MCH	28.4 pg	28.8 pg	-	27 – 34
Platelets	13 10^9/L	27 10^9/L	6 10^9/L	150 – 450
BUN	18.7 mg/dL	33.2 mg/dL	40 mg/dL	8 – 23
GPT	16 U/L	-	-	0 – 45
GOT	21 U/L	-	-	0 – 40
Creatinine	1.22 mg/dL	1.01 mg/dL	1.06 mg/dL	0.72 – 1.18
Glucose	80 mg/dL	117 mg/dL		74 – 106
Complement C3	99 mg/dL	-	-	90 – 180
Complement C4	27.6 mg/dL	-	-	10 – 40
Fibrinogen	227 mg/dL	-	-	200 - 400
IgE	556.3 UI/mL	-	-	0 – 100
IgA	72 mg/dL	-	-	70 – 400
IgG	419 mg/dL	-	-	700 – 1600
IgM	72 mg/dL	-	-	40 - 230
RF	< 8 UI/mL	-	-	Until 8
ANAs	Negative	-	-	Negative
Anti-dsDNA	Negative	-	-	Negative
TSH	0.67 mIU/L	-	-	0.4 – 4
T4	0.79 ng/dL	-	-	0.89 – 1.76
Prothrombin time	11 seconds	11 seconds	11 seconds	9 - 13
Partial Thromboplastin Time	31 seconds	25 seconds	22 seconds	25 - 35
D-dimer	-	7.45 mg/L	-	0 - 0.50

ANA = anti-nuclear antibodies; Anti-dsDNA = anti-double stranded DNA; BUN = blood urea nitrogen; GOT = glutamic-oxaloacetic transaminase; GPT = glutamic-pyruvic transaminase; Ig = immunoglobulin; MCH = mean corpuscular hemoglobin; MCHC = mean corpuscular hemoglobin concentration; MCV = mean corpuscular volume; RF = rheumatoid factor; TSH = thyroid stimulating hormone.

In the ICU, the patient’s clinical condition deteriorated with a marked decrease in platelets, anemia, and hypotension. Vasopressor therapy was then started, along with platelet and red blood cell transfusions as needed. A positive Coombs test led the hematology team to consider therapy with dexamethasone and azathioprine. An evaluation by the infectious disease service was requested due to suspicion of rickettsiosis. Empirical doxycycline was initiated, but rickettsiosis was subsequently ruled out. An ITP diagnosis, possibly triggered by multiple tick bites, was also suggested. Despite therapy, on January 19^th^ the patient’s condition continued to deteriorate, with evidence of melena, leukocytosis associated with corticosteroid therapy, anemia, thrombocytopenia, and lactic acidosis with persistent hypotension. Unfortunately, on January 20^th^ the patient experienced three episodes of cardiopulmonary arrest and died. An autopsy was conducted, and histopathological examination of a liver sample revealed no inflammatory infiltrate, nor any suggestive findings of malaria, yellow fever, or spotted fever group rickettsiosis (by immunohistochemistry). Ticks were collected from the pastures where the patient had been working, resulting in 281 specimens which were classified as *Amblyomma* spp. larvae (Figure 1D).

### Ethics

Written informed consent was obtained from the patient.

## DISCUSSION

ITP pathogenesis involves the generation of anti-platelet autoantibodies which triggers bleeding lesions due to the improper formation of thrombi, clinically manifested as petechiae, ecchymoses, hematomas, and gingival bleeding^1^. Blood in the urine or stool are characteristics that often indicate profuse internal bleeding, which portends a worse clinical outcome^1^. ITP is usually an exclusion diagnosis; in our case, common thrombocytopenia causes were excluded due to low diagnostic probability and negative diagnostic tests. Despite not fitting the clinical profile of dengue (early severe thrombocytopenia without leukopenia and no other symptoms), HIV infection, leptospirosis (timeline discordant with natural history), or malaria (there was no anemia or splenomegaly to suggest a sequestration phenomenon), diagnostic testing was still conducted for these diseases yielding negative results. The above left us with two possibilities: rickettsiosis (with low probability given the absence of fever, electrolyte compromise, and a timeline not typical for complications) and secondary ITP in temporal relation to tick bites.

Although most common in children, acute ITP can also occur in adults^1^ and while uncommon, arthropod bites can trigger acute ITP. Four acute ITP cases due to bee stings and insect bites^2-4^ have been reported in the literature, as well as an ITP case following honeybee-venom therapy^5^. Although not fully understood, it seems that the active components of bee venom can trigger severe hematological and immune reactions^6^. However, to our knowledge, no ITP cases due to tick bites have been reported.

## CONCLUSION

Although ticks are not venomous arthropods, their saliva contains several components that are homologous to proteins found in scorpions, spiders, and bees, suggesting that tick saliva could trigger an immune and inflammatory response similar to other arthropod venoms^7^. Conversely, tick saliva contains many proteins from various protein families that display antihemostatic activities^8^. The largest group comprises serine protease inhibitors that target thrombin, activated coagulation factor X, and other proteases from the coagulation cascade^8^. Platelet aggregation is inhibited by tick proteins that either bind to platelet integrins, scavenge platelet activators (e.g., adenosine diphosphate, thromboxane, or serotonin) or inhibit the activation of protease-activated receptors^8^. As the patient suffered multiple tick bites, the large amount of saliva inoculated could have triggered the disease. Given the above, hyperinfestation by ticks could be considered an additional ITP cause due to arthropod bites.
